# Differential neural activity predicts the long-term stability of the effects of positive and negative expectations on pain

**DOI:** 10.1038/s41598-024-77693-z

**Published:** 2024-11-13

**Authors:** Maren-Isabel Wolf, Christoph Arne Wittkamp, Michael Rose

**Affiliations:** Department of Systems Neuroscience, University Medical Center Hamburg-Eppendorf, Hamburg, Germany

**Keywords:** Neuroscience, Placebo effect, Human behaviour

## Abstract

Expectations modulating pain perception is a well-researched phenomenon, but less is known about the persistence of expectation effects over longer time-courses. In this preregistered study, we examined the persistence of positive (placebo) and negative (nocebo) expectation effects over one week and investigated whether neural activity on day 1 (fMRI) can predict the stability of these effects one week later (*n* = 41). We tested whether expectations were reflected in EEG oscillatory activity at the second measurement. Both positive and negative pain modulation effects persisted over the tested time-period and did not undergo extinction. Expectations of higher compared to lower pain led to larger theta-to-alpha EEG activity. Most interestingly, differential neural activity in fMRI was correlated with persistent expectations. Individual differences in the persistence of positive expectation effects were related to reduced amygdala activity and enhanced activity in the anterior insula and dorsolateral prefrontal cortex (DLPFC) during the first session. In contrast, persistence of negative expectation effects was predicted by enhanced thalamus activity. Our findings indicate relatively stable placebo and nocebo effects over longer time courses, but this persistence is based on different neural areas for positive and negative expectations.

**Supplementary Information:**

The online version contains supplementary material available at 10.1038/s41598-024-77693-z.

## Introduction

Expectations can influence the perception of pain and thereby lead to hypoalgesia (placebo effect) or hyperalgesia (nocebo effect)^1^. These perceptual modulations are thought to be instantiated through activation and deactivation of the so-called descending pain modulatory system on the neural level (DPMS)^2,3^, leading to measurable differences in the processing of painful stimuli^1,4^. However, few studies so far investigated the stability of these expectation effects across longer time periods^5–7^.

In a standard Bayesian or non-Bayesian learning model, placebo and nocebo effects would be expected to decrease after the offset of reinforcement and disappear over time, as participants would update their beliefs when continuously receiving stimuli that contradict their expectations^8,9^. In spite of this, recent behavioral and neural evidence suggests that expectations can work via self-reinforcing feedback loops that prevent them from extinction^8,9^. This fits with evidence of highly stable placebo effects in clinical studies^10–14^, while controlled experimental research on the stability of expectation effects over multiple days is sparse but much needed to gain mechanistic insights into these effects^15^. Within one session, both placebo hypoalgesia and nocebo hyperalgesia have shown to be relatively stable over multiple test trials^8,9,16–22^, depending e.g. on the number of conditioning stimuli^23^ and the valence of expectations^18,21^. Regarding the valence of expectations, there is some evidence for a higher persistence of nocebo compared to placebo effects, possibly due to higher arousal that impedes learning from experiences in nocebo groups^18,21^. Additionally, it has been demonstrated that partial reinforcement during conditioning is more effective in creating stable placebo effects over a test phase than consistent reinforcement^24^. Few studies examined effects over longer time-courses. For example, Whalley et al. induced placebo effects using only verbal instructions and observed similarly high effects in two sessions between one and eight days apart^5^. In contrast, Colloca et al. found a slight decrease of placebo effects after four to seven days, although importantly, effects were still evident in the second session^19^. Regarding negative expectations, a nocebo effect in tactile perception was still detectable one week later^25^, while to our knowledge there has not been any investigation on the consistency of nocebo responses in pain perception after multiple days.

The conceivable persistence of placebo and nocebo effects in pain perception further raises the crucial question of which neuronal areas predict the persistence of expectations over a longer period. Considering that the prefrontal cortex might be responsible for the neural suppression of learning from prediction errors in placebo hypoalgesia^8^, the persistence of expectations might be connected to this area. Beyond this, activity in other areas that give rise to the expectation-related modulation of pain might mediate the stability of placebo and nocebo effects, such as areas of the DPMS like the dorsolateral prefrontal cortex (DLPFC), ventromedial prefrontal cortex (vmPFC), or the anterior cingulate cortex (ACC)^2^, as well as the insula^26,27^. Interestingly, we have previously demonstrated that many of these areas were similarly activated for both positive and negative expectations during pain anticipation, while activation in the same regions differentiated during pain perception^28^. Likewise, predictive activity for persistent expectation effects might differ between pain anticipation and pain perception. Moreover, it is unclear whether predictive activity for upholding positive and negative expectations can be found in similar or different areas, when considering findings on the nocebo effects being more easily induced and more enduring compared to placebo effects^18,21^.

**Fig. 1:**
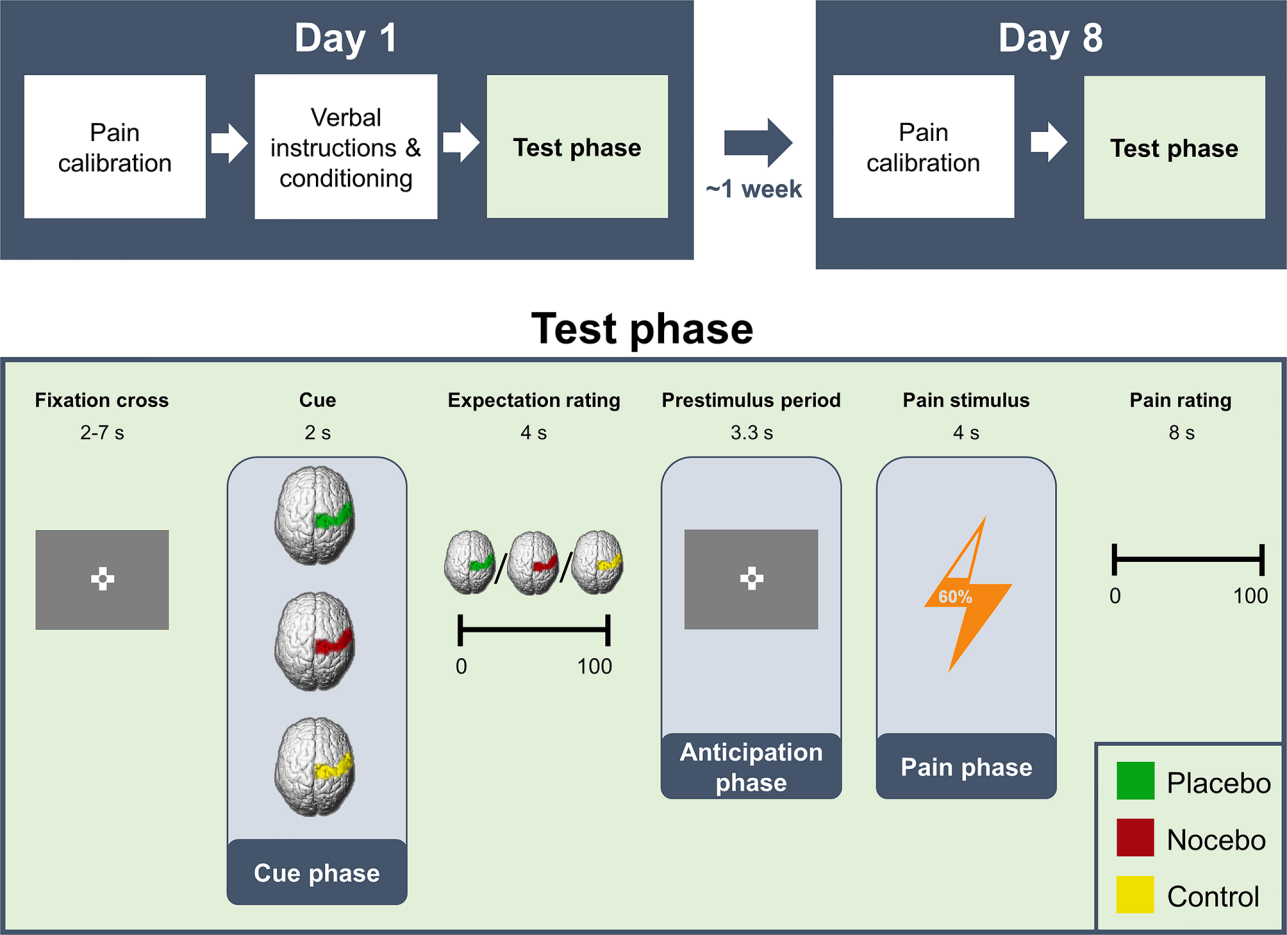
Experimental structure. The experiment consisted of two sessions approximately one week apart (day 1 and day 8). On day 1 only, positive and negative pain-related expectations regarding visual cues were induced using verbal instructions and a conditioning procedure. On both day 1 and day 8, a similar test phase was performed, in which participants always received the same pain intensity, but condition cues varied from trial to trial. Pain intensities were individually calibrated on both days.

With this study, we aimed to induce both positive and negative expectations to elicit placebo hypoalgesia and nocebo hyperalgesia in healthy participants, with the main objective to test the stability of the effects after one week. Further, we investigated neural predictors of the stability of effects. Lastly, we aimed to assess whether there were any differences in neural activity during the anticipation and pain phase between positive and negative expectations on day 8, measured by EEG oscillatory activity. We examined participants that underwent a conditioning procedure and verbal instructions inducing positive and negative expectations in one session (day 1), while being subjected to a combined fMRI and EEG measurement. Participants were then re-invited to the lab roughly one week later for a second test session (day 8), undergoing EEG measurement only. In this second session, we performed an identical procedure, but without a conditioning phase or verbal instructions to reinstate expectations (see Fig. 1). Due to the change in the external environment from day 1 (MR lab) to day 8 (EEG lab), we were also able to evaluate whether expectation effects would remain stable despite a change in the “treatment” context. Previously, we have shown that expectations were reliably induced on day 1 and were reflected in common neural activity for positive and negative expectations before the painful stimulus was applied but differentiated during pain stimulation^28^. Here, we focused on the stability of the behavioral effects over one week, more specifically, how stable placebo and nocebo effects were over the time course of one week, and how the stability could be predicted using behavioral and neuronal markers.

Based on previous findings, we expected to find persisting placebo and nocebo effects after one week on the behavioral level^5,8,9,19^. As positive and negative expectations have shown to affect EEG oscillatory activity, especially when comparing expectations of high vs. low pain, we also expected to see differences in EEG activity between placebo and nocebo on day 8^29–31^. Lastly, we expected that the strength of behavioral effects on day 8 could be predicted by the magnitude of placebo- and nocebo-related fMRI activity on day 1, more specifically, in areas commonly connected to expectation effects such as parts of the DPMS. As previous research has demonstrated different patterns of activity in the anticipation phase preceding the application of the stimulus compared to the actual pain phase, we aimed to identify predictive effects within both of these periods.

## Results

To test for the stability of expectation effects, 41 participants (26 female) came to the lab twice, approximately one week apart (see Fig. 1). On day 1, participants received sham feedback regarding their current pain sensitivity in the form of a colored cue, that was supposedly calculated by a brain-computer interface algorithm, to induce either placebo hypoalgesia, nocebo hyperalgesia or no directed expectation (control) while being subjected to EEG and fMRI measurements (for more information see Methods). Participants also underwent a conditioning procedure in which the placebo cue was paired with 10 lowly painful stimuli (VAS 30) and the nocebo cue was paired with 10 highly painful stimuli (VAS 70) on day 1. In the subsequent test phase, participants received feedback regarding their supposed pain sensitivity on each trial, had to indicate how painful they would expect to perceive the next stimulus, and after being presented with the same pain stimulus in every trial (VAS60) had to rate the actual perceived intensity. An identical test phase was conducted on day 8 while undergoing EEG measurement with the difference that no conditioning or verbal suggestion took place. Further details regarding this procedure and findings regarding the combined EEG-fMRI measurement on day 1 have been published elsewhere^28^.

### Induction and stability of effects

The successful induction of effects on day 1 has already been reported elsewhere^28^. In brief, expectation and pain ratings were significantly modulated by the three condition cues on day 1, leading to higher ratings for the nocebo compared to the control and in turn for the control condition compared to the placebo condition.

**Fig. 2:**
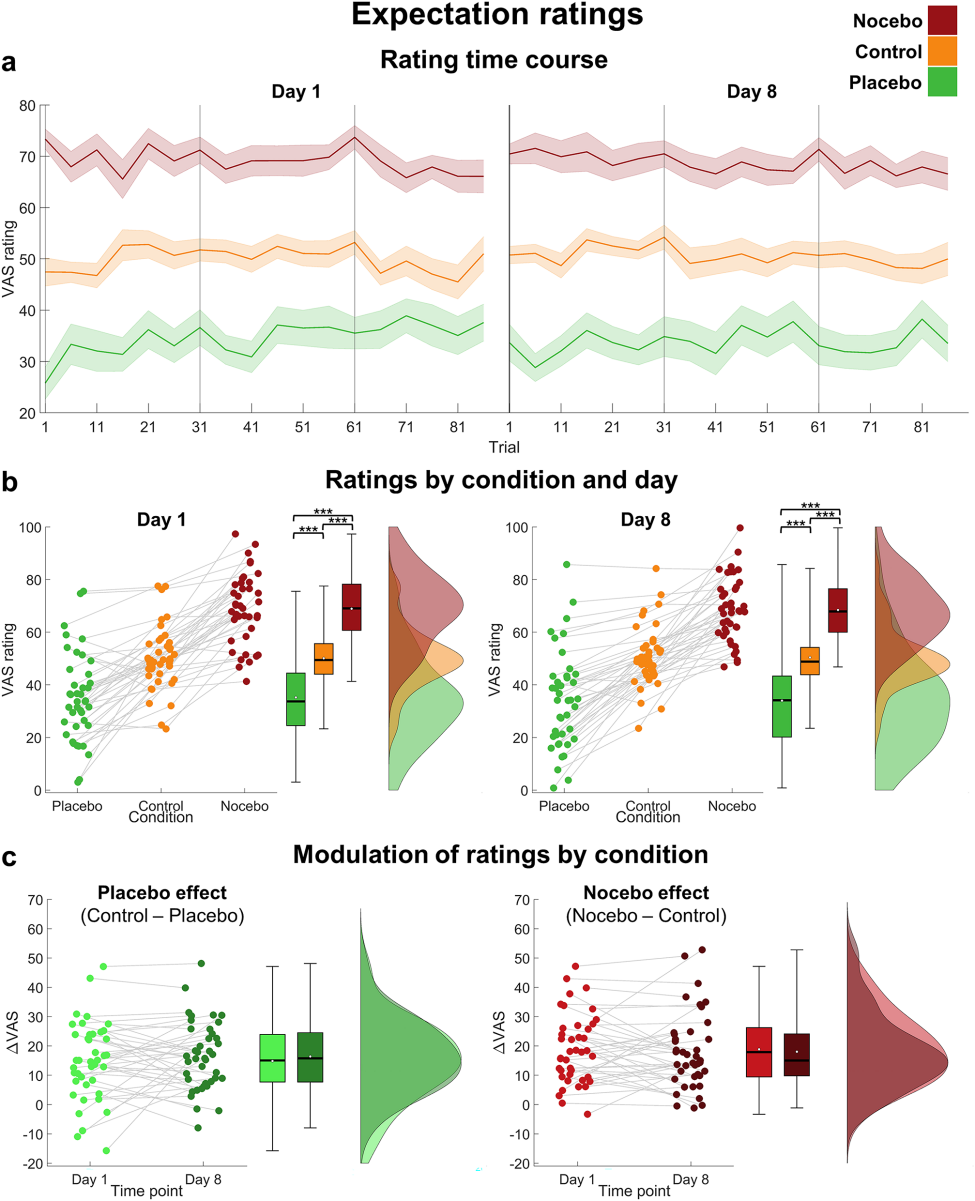
Expectation ratings. (**a**) Time course of expectation ratings per condition, averaged over five trials. Error bars denote the corrected standard error of the mean (SEM) using the Cousineau-Morey method^32,33^. (**b**) Raincloud plots^34^ of expectation ratings per condition on day 1 (left) and day 8 (right). Each dot represents the mean rating of an individual subject per condition and grey lines connect the ratings of the same subject over conditions. The black line inside the boxplots shows the median, the white dot depicts the mean. (**c**) Placebo effect (difference between the control and placebo condition; left) and nocebo effect (difference between the nocebo and control condition; right) in expectation ratings per day. ***p < .001.

**Fig. 3:**
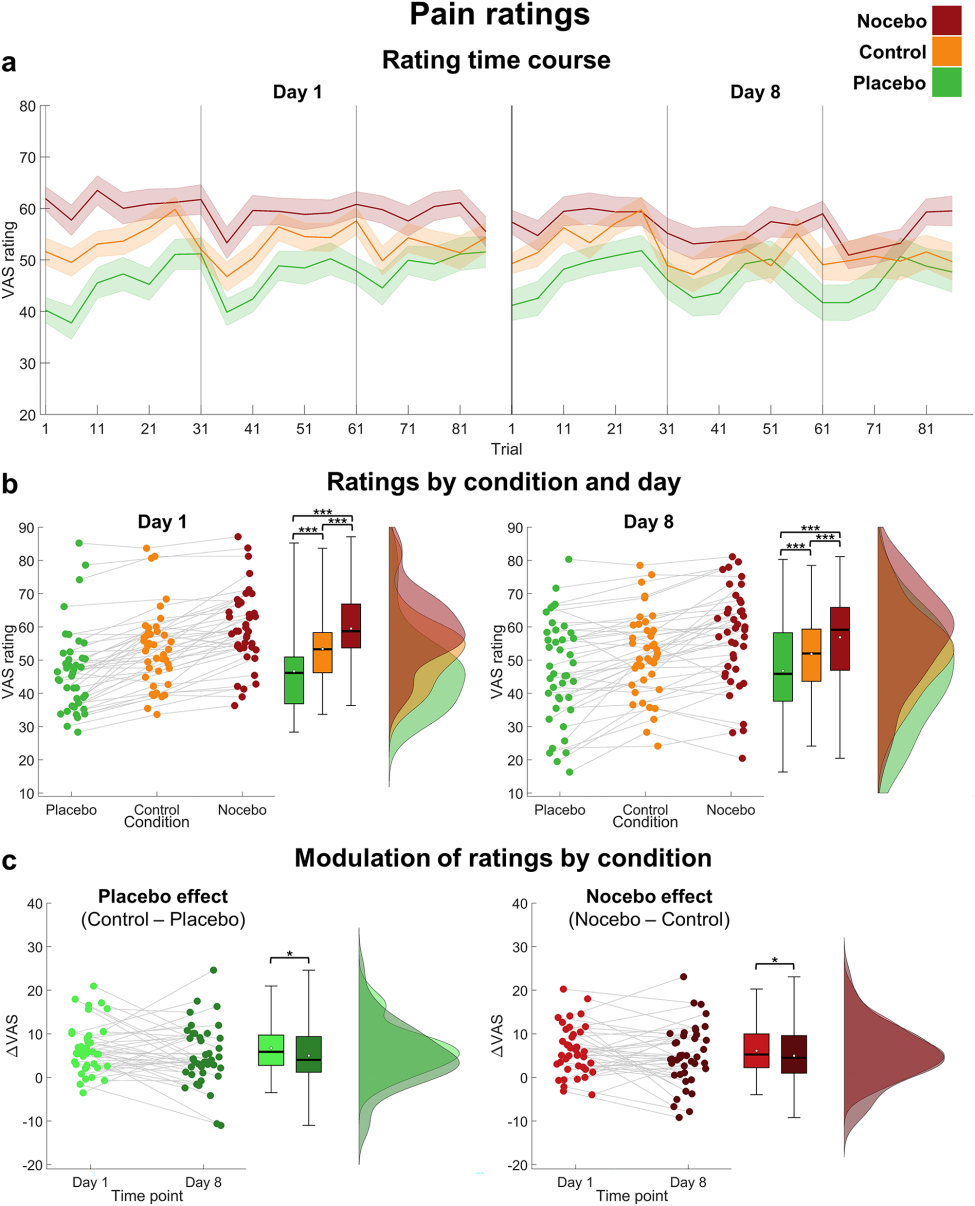
Pain ratings. (**a**) Time course of pain ratings per condition, averaged over five trials. Error bars denote the corrected standard error of the mean (SEM) using the Cousineau-Morey method^32,33^. (**b**) Raincloud plots^34^ of pain ratings per condition on day 1 (left) and day 8 (right). Each dot represents the mean rating of an individual subject per condition and grey lines connect the ratings of the same subject over conditions. The black line inside the boxplots shows the median, the white dot depicts the mean. (**c**) Placebo effect (difference between the control and placebo condition; left) and nocebo effect (difference between the nocebo and control condition; right) in pain ratings per day. *p < .05. ***p < .001.

To investigate whether the induced effects were still evident one week later, we performed repeated measures ANOVAs with pain and expectation ratings as outcomes and included the time point (day 1, day 8) additionally to the condition (placebo, nocebo, control) as factor (descriptive time courses of expectation and pain ratings per condition and day are shown in Figs. 2a and 3a).

Expectation ratings were significantly affected by the condition, but there was no significant effect of time point and no interaction effect, indicating that there were no differences between day 1 and day 8 (see Table 1; Fig. 2b). Over both days, expectation ratings were higher in the nocebo (*M* = 68.66, *SD* = 11.25) compared to the control condition (*M* = 50.23, *SD* = 10.91, *p*_holm_ < 0.001), which in turn were higher than in the placebo condition (*M* = 34.60, *SD* = 15.91, *p*_holm_ < 0.001).

**Table 1:** Results of a repeated measures ANOVA for expectation and pain ratings with condition (placebo, nocebo, control) and time point (day 1, day 8) as factors.

	Expectation ratings				Pain ratings			
	df	F	*p*	η²_*p*_	df	F	*p*	η²_*p*_
Condition	1.14^a^	112.68	< 0.001	0.74	1.13^a^	63.15	< 0.001	0.61
	45.45^a^				50.98^a^			
Time point	1.00^a^	0.13	0.724	< 0.01	1.00^a^	0.58	0.451	0.01
	40.00^a^				40.00^a^			
Condition ✻ Time point	1.22^a^	0.21	0.700	< 0.01	2.00^a^	2.89	0.061	0.07
	48.63^a^				80.00^a^			

^a^The degrees of freedom were corrected using Greenhouse-Geisser estimates of sphericity as Mauchly’s test of sphericity indicated that the assumption of sphericity was violated (*p* < .05).

Similarly, pain ratings were significantly affected by the condition, but not by the time point nor their interaction (see Table 1; Fig. 3b). Pain ratings were higher in the nocebo (*M* = 58.19, *SD* = 11.46) compared to the control condition (*M* = 52.63, *SD* = 10.70, *p*_holm_ < 0.001), which in turn led to higher ratings than the placebo condition (*M* = 46.75, *SD* = 12.47, *p*_holm_ < 0.001). Thus, both pain and expectation ratings were significantly modulated by the condition cue in line with our manipulation and this effect appeared to be stable between day 1 and day 8. Moreover, the non-significant effect of time point hints that pain perception over all three conditions was stable over both measurement days, indicating that participants subjectively perceived pain stimuli on both days as equally painful.

We further compared the amount of variability in individual ratings between conditions and days. For expectation ratings, we found significant differences in the relative variability index (see Methods section for details) between conditions (*F*(2,80) = 9.99, *p* < .001), but no differences between days. Post-hoc tests revealed that there was less variability in the control condition compared to both placebo and nocebo condition (both *p*_holm_ < 0.05). In contrast, variability in pain ratings did not significantly differ between conditions, but increased from day 1 to day 8 (*F*(1,40) = 13.45, *p* < .001; post-hoc test day 1 vs. day 8: *p*_holm_ < 0.001). These results indicate that expectation ratings in the control condition might have been more driven by the cue compared to the other conditions, which was not the case in the pain ratings. Further, the similar level of variability in pain ratings across all three conditions indicated a dynamic updating of expectations.

Next, we assessed the stability of placebo and nocebo effects separately. *Placebo effects* (control minus placebo) and *nocebo effects* (nocebo minus control) were calculated as absolute differences in a manner that ensured that a higher rating modulation in the intended direction was always indicated by higher scores.

For expectation ratings, a rmANOVA revealed no differences in rating modulation between day 1 and day 8, no differences between placebo and nocebo effects and no interaction, implying no reduction in neither placebo nor nocebo effect between day 1 and day 8 (see Fig. 2c; Table 2). In contrast, pain rating modulations significantly differed between day 1 and day 8 (see Fig. 3c; Table 2). Post-hoc tests showed a significant decrease in pain rating modulation from day 1 (*M* = 6.43, *SD* = 4.30) compared to day 8 (*M* = 5.01, *SD* = 5.25, *p*_holm_ = 0.036), indicating that both placebo and nocebo effects were stronger on day 1 compared to day 8.

Although placebo and nocebo effects on pain ratings appeared to decrease over time, they were still highly evident on day 8 (placebo effect: *t*(40) = 4.65, *p* < .001, *d* = 0.73; nocebo effect: *t*(40) = 4.68, *p* < .001, *d* = 0.73), just like expectation rating effects were (placebo effect: *t*(40) = 9.00, *p* < .001, *d* = 1.41; nocebo effect: *t*(40) = 8.95, *p* < .001, *d* = 1.40). Most strikingly, this was still true at the final block of day 8 for both expectation (placebo effect: *t*(40) = 7.94, *p* < .001, *d* = 1.24; nocebo effect: *t*(40) = 8.55, *p* < .001, *d* = 1.34) and pain ratings (placebo effect: *t*(40) = 3.17, *p* = .001, *d* = 0.50; nocebo effect: *t*(40) = 3.18, *p* = .001, *d* = 0.50).

**Table 2:** Results of a repeated measures ANOVA for expectation and pain ratings with modulation type (placebo effect, nocebo effect) and time point (day 1, day 8) as factors.

	Expectation ratings				Pain ratings			
	df	F	*p*	η²_*p*_	df	F	*p*	η²_*p*_
Modulation type	1	3.95	0.054	0.09	1	0.13	0.722	< 0.01
	40				40			
Time point (TP)	1	0.06	0.816	< 0.01	1	4.70	0.036	0.11
	40				40			
Modulation type ✻ TP	1	1.62	0.211	0.04	1	0.10	0.760	< 0.01
	40				40			

Interestingly, on the descriptive level, placebo and nocebo effects appeared to be even larger in the first trials of day 8 compared to the last trials of day 1 (see Figs. 2a and 3a). This observation was corroborated by an exploratory statistical comparison of placebo and nocebo effects in the last five trials of each condition on day 1 to the first five trials of each condition on day 8 (see Table 3). Post-hoc tests revealed an increase in expectation rating modulation from the last five trials of day 1 (*M* = 14.63, *SD* = 13.95) to the first five trials of day 8 (*M* = 19.36, *SD* = 10.64; *p*_holm_ = 0.006) for both placebo and nocebo effect. Similarly, pain rating modulations were stronger at the start of day 8 (*M* = 6.33, *SD* = 6.90) compared to the end of day 1 (*M* = 4.12, *SD* = 4.73; *p*_holm_ = 0.031).

**Table 3:** Results of a repeated measures ANOVA for expectation and pain ratings with modulation type (placebo effect, nocebo effect) and time point (last 5 trials day 1, first 5 trials day 8) as factors.

	Expectation ratings				Pain ratings			
	df	F	*p*	η²_*p*_	df	F	*p*	η²_*p*_
Modulation type	1	3.46	0.070	0.08	1	0.51	0.482	0.01
Residuals	40				40			
Time point (TP)	1	8.60	0.006	0.18	1	5.01	0.031	0.11
Residuals	40				40			
Modulation type ✻ TP	1	2.52	0.120	0.06	1	0.97	0.331	0.02
Residuals	40				40			

As the inter-test period was intended to span 7 days but exhibited slight variations between participants (see Methods), we tested whether the actual interval affected the stability of placebo and nocebo effects. There was no significant relationship of the inter-test period with placebo (expectation ratings: *r* = .23, *p* = .153; pain ratings: *r* = -.03, *p* = .851) or nocebo effects on day 8 (expectation ratings: *r* = .11, *p* = .478; pain ratings: *r* = -.29, *p* = .061). Furthermore, there was no significant relationship of the inter-test period with the change in placebo (expectation ratings: *r* = .04, *p* = .804; pain ratings: *r* = -.09, *p* = .591) and nocebo effects (expectation ratings: *r* = .01, *p* = .970; pain ratings: *r* = -.14, *p* = .930) from day 1 to day 8 (calculated as the absolute difference between effects on day 1 and day 8).

### EEG activity

**Fig. 4:**
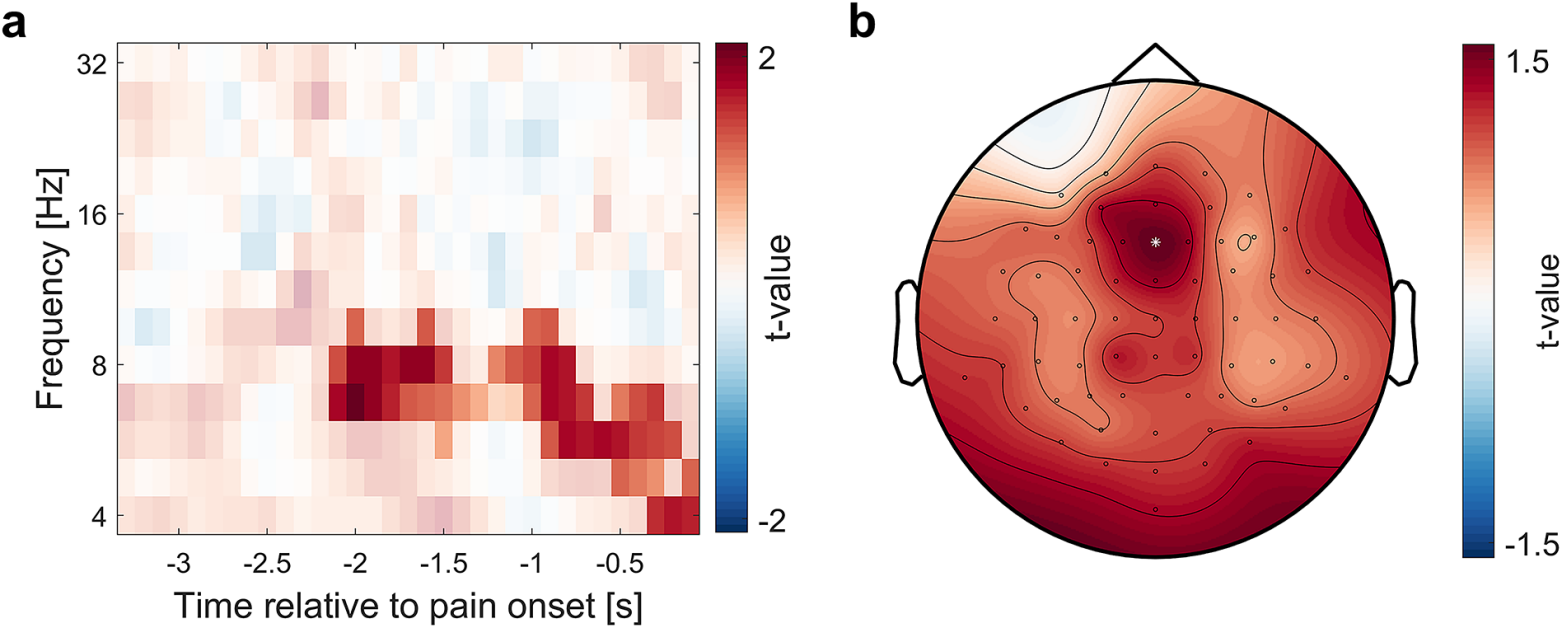
Differences in EEG power between placebo and nocebo on day 8. (**a**) Time-frequency plot of *t*-values for placebo vs. nocebo in the anticipation phase on day 8 averaged over all cluster electrodes. (**b**) The corresponding topography (peak electrode Fz highlighted with a white star).

To assess whether differences in ratings on day 8 were accompanied by changes in brain activity, we compared EEG power between the nocebo and placebo condition. There were significant cluster-corrected differences in theta-to-alpha power during the anticipation phase (-2.1 until − 0.1 s, 4 to 9.5 Hz; *p* = .025, see Fig. 4), suggesting that the expectation effects were not only stable on the behavioral level, but also evident in enhanced low frequency power for nocebo compared to placebo on the neural level. During the pain phase, oscillatory power did not significantly differ between the placebo and the nocebo condition. We further compared EEG power to detect placebo- (placebo vs. control) or nocebo-specific (nocebo vs. control) differences in oscillatory power. There were no significant cluster-corrected differences for these comparisons (all *p* > .05).

### Relation of behavioral effects across days

**Fig. 5:**
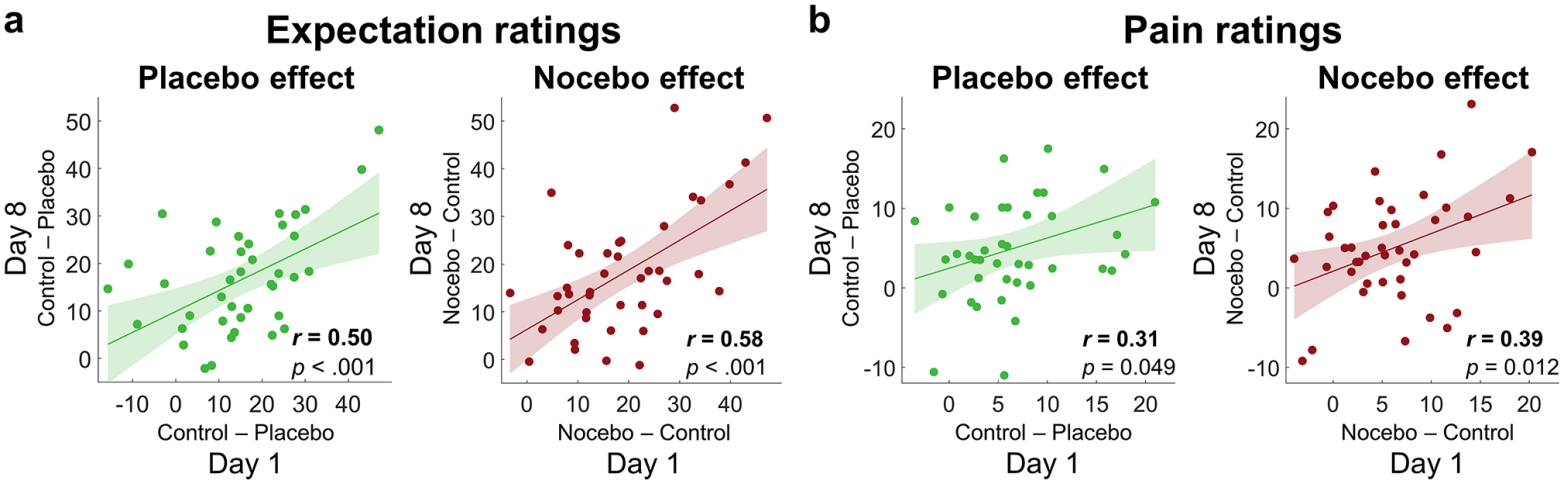
Prediction of effects on day 8 based on the effects observed on day 1. Correlation plots for expectation (**a**) and pain ratings (**b**), showing the relationship between the individual placebo and nocebo effects on day 1 with the corresponding effects on day 8. Each dot represents one subject.

To determine the relation of behavioral placebo and nocebo effects over the measurement days, we correlated placebo and nocebo effects on day 1 with the corresponding effects on day 8. For both expectation and pain ratings, the strength of individual placebo and nocebo effects on day 8 were correlated with the corresponding effects on day 1 (see Fig. 5, all *p* < .05), indicating that effects were not only stable on the group level, but that the individual strength of effects on day 8 was largely determined on how strong effects were on day 1.

### Neural correlates of the persistence of placebo and nocebo effects

Next, we assessed whether the neuronal processing on day 1 could predict the persistence of behavioral placebo and nocebo effects. For this aim, we used the individual strength of placebo and nocebo effects on pain ratings on day 8 and tested for a relation with the fMRI activity on day 1 (corrected for the strength of the behavioral effect on day 1, see Methods). We tested for predictive fMRI activity both in the time frame directly prior to the application of the pain stimulus (anticipation phase) and during pain processing itself (pain phase; see Fig. 1 for details regarding the trial structure).

**Fig. 6:**
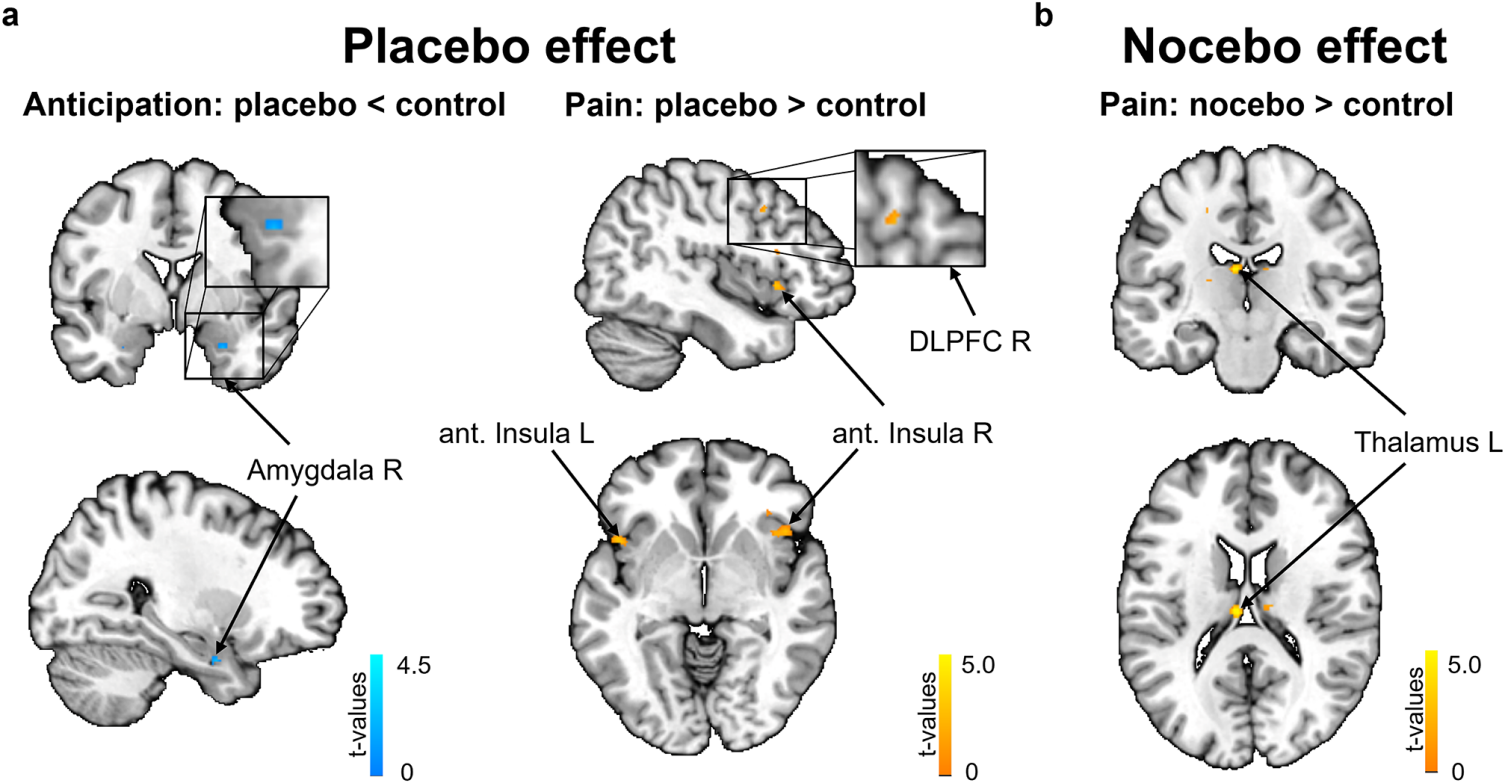
Neural predictors of placebo and nocebo effect persistence. (**a**) Analyses of the persistence of expectation effects using individual placebo effects of day 8; Blue: lower activation for the placebo condition compared to the control condition in the amygdala during the anticipation phase; Yellow: higher activation in the insula and DLPFC during pain perception in the comparison to control. **(b)** Analyses of the persistence of expectation effects using individual nocebo effects of day 8; Yellow: higher activation in the thalamus during pain perception in the comparison of nocebo to control.

Individual differences in the placebo effect on day 8 revealed activity differences in the comparison of the placebo condition to the control condition (for detailed results see Table 4). Participants showing higher placebo effects on day 8 showed activity reductions in the amygdala during the anticipation of pain and higher activation in the left and right anterior insula and the right DLPFC during pain perception on day 1 (see Fig. 6a). Analyzing individual differences in the nocebo effect revealed higher activation in the thalamus in subjects showing larger nocebo effects on day 8 in the comparison of the nocebo condition to the control condition during pain perception on day 1 (see Fig. 6b). Additional analyses in other ROIs (see Methods) yielded no significant effects.

**Table 4:** Peak coordinates and statistics of regions for analyses of the predictive power of brain areas for the persistence of expectation effects, using individual differences in the placebo and nocebo effects on day 8 (controlled for the behavioral effect of day 1, see Methods).

Region		MNI Coordinates				
	Hemi.	X	Y	Z	t	*p*_FWE_
**Placebo Effect as Covariate**:						
Placebo < Control during the Anticipation Phase						
Amygdala	R	28	0	-26	4.19	0.021†
Placebo > Control during the Pain Phase						
Anterior Insula	R	40	18	-2	4.85	0.021†
	L	-42	12	-6	4.60	0.040†
DLFPC	R	44	12	40	4.21	0.048†
**Nocebo Effect as Covariate**:						
Nocebo > Control during the Pain Phase						
Thalamus	R	-6	-18	18	4.80	0.016†

Coordinates are in MNI space. DLPFC = Dorsolateral Prefrontal Cortex. Hemi = Hemisphere. †small-volume corrected.

## Discussion

Examining the persistence of previously induced placebo and nocebo effects over the time-period of one week enabled the assessment of their temporal stability. Both placebo and nocebo effect were revealed to be relatively persistent after one week, even though there was a significant decrease in the strength of both placebo and nocebo effects in pain ratings. However, both effects showed a rebound of effect strength from the last trials of day 1 to the first trials of day 8 and underwent no extinction over the entire time course of the experiment. The comparison of EEG oscillatory activity for nocebo vs. placebo revealed that expectations were represented differentially in anticipatory theta-to-alpha activity on day 8. The persistence of effects was largely dependent on the individual strength of placebo and nocebo effects on day 1, as participants that showed greater effects on day 1 also showed greater effects on day 8. We further investigated the neuronal correlates of the persistence of effects and found that a stronger persistence of placebo effects was predicted by larger placebo-induced modulation of fMRI activity in the amygdala during the anticipation of pain and in the right DLPFC and the bilateral anterior insula during pain processing on day 1. Conversely, the persistence of nocebo effects was correlated with larger nocebo-induced changes in fMRI activity in the thalamus during pain perception.

Our finding of relatively stable placebo and nocebo effects, both on the group and individual level, contribute to the existing body of knowledge on the highly persistent nature of placebo and nocebo effects in clinical settings^10^ and expands the understanding of the longer-term stability of placebo effects^5,19^. Importantly, we used a larger number of test trials per session than most previous studies^19–21,23^, which led to participants constantly receiving sensory information that did not fit their beliefs on the objective level. Nevertheless, placebo and nocebo effects did not undergo extinction. Thus, our results further challenge classical learning models in the context of placebo and nocebo effects, which would predict that participants learn from their experiences and adjust their expectations over time, leading to a reduction of placebo and nocebo effects^8,9^, but highlight the need for more complex interpretations. One possible mechanism is self-reinforcing feedback loops, whereby participants tend to learn more from experiences that align with their expectations, leading to a stabilization of these expectations so that they can withstand potentially invalidating information^8,9^. At least within one session, this may explain the stability of effects.

It is important to note that the placebo and nocebo effects stayed stable between sessions and even increased between the last trials of day 1 and the first trials of day 8. The effects were further found to persist after the change in treatment context from day 1 (MRI lab) to day 8 (EEG lab), in line with previous findings^5^. This suggests that the induced beliefs regarding the efficacy of our treatment were not overwritten by the experience on day 1 but showed a rebound in strength. Other potential explanations for the lack of decrease in expectations include that our combination of verbal instructions and strong conditioning induced highly persistent initial beliefs^23,35^, or that there was a shift in expectations from being driven by beliefs to more unconscious associations over time^10^. However, the effects on expectation ratings were remarkably stable, which indicates that participants were aware of their beliefs, suggesting rather a conscious representation of expectations. As it has already been demonstrated that both conditioning^36,37^ and verbal instructions alone^5,38^ can lead to reliable placebo and nocebo effects, further studies could compare the long-term stability of more conscious (i.e. verbal instructions) versus unconscious methods of expectation induction (i.e. conditioning) to elucidate different factors that might affect the stability of expectation effects.

We observed no difference between placebo and nocebo effects both in terms of strength and stability. This is somewhat surprising given previous evidence suggesting that nocebo effects can be introduced more easily and are more robust against extinction^18,21^. Nevertheless, it has to be considered that the majority of these findings were derived from studies that investigated the course of expectations in one session only^18,20,21^. To our knowledge, we are the first to examine the longer-term stability of negative pain-related expectations. The persistence of both placebo and nocebo effects may be related to the high number of conditioning stimuli that we used (10 per condition), as the number of conditioning stimuli is related to the strength of induced effects^23^. The differential stability of placebo and nocebo effects within shorter time frames has been attributed to higher arousal in nocebo compared to placebo blocks, which impedes learning from experiences and thereby stabilizes nocebo effects^18^. Here, conditions within blocks were pseudo-randomized, which could lead to a similarly high level of arousal for all conditions and therefore similarly low rates of extinction in both placebo and nocebo effects.

Interestingly, we found predictive neural activity for the stability of both placebo and nocebo effects. For placebo effects, this activity was located in areas associated with the DPMS during pain anticipation in the amygdala, and during pain processing in the DLPFC and the anterior insula. The DPMS is proposed to play a pivotal role in placebo and nocebo effects^2,3^. The amygdala exhibits direct connections to classical placebo areas like the vmPFC and the periaquedactual grey (PAG)^2^ and decreased activity in the amygdala has been associated with increased placebo effects^4^. It further has been reported to show predictive activity for participants perceiving stimuli as more painful despite receiving an explicitly neutral cream^39^. Additionally, a rightward asymmetry in the volume of subcortical limbic structures including the amygdala has been reported for placebo responders compared to non-responders^40^. These findings underline the importance of this structure for the placebo response and the individual differences in response quality. This could be mediated by the important role the amygdala has in threat assessment^41^. A reduced threat perception might lead to higher and more stable placebo effects. As the amygdala is a key structure in associative learning^2,42,43^, reduced activity could also be interpreted as a reduction in learning from experiences during the test phase, leading to more pervasive placebo effects.

The DLPFC has been linked to placebo effects in both anticipation of pain and during pain perception^26–28,44,45^. The DLPFC has a pivotal role in the top-down modulation of placebo effects^2^. This could possibly relate to the suppression of learning from prediction errors described by Schenk et al.^8^. Moreover, activity in the DLPFC has been reported to have predictive power for individual placebo responses^44^. In conjunction with our findings, this illustrates the importance of the DLPFC in not only predicting individual placebo responses, but also the individual persistence of these responses. The anterior insula also has often been reported to show increased activation in placebo conditions^2^ and further has been reported to have predictive power for individual placebo responses^44^. It also has been marked it as an important node in the salience network^46^, and it may also play an important part in evaluating the expected threat level^28,47,48^. Considering the involvement of the amygdala and the anterior insula in affective processing and associative learning, the persistence of placebo effects might depend on the affective evaluation of the stimuli and learning processes triggered by it, with activity in the amygdala mediating this evaluation during the anticipation of pain and the anterior insula mediating the evaluation during pain perception. This effect of the affective evaluation might be extended by a strong top-down modulation by the DLPFC^8^.

The persistence of nocebo effects was predicted by increased activity in the thalamus. The thalamus has been reported to encode nociceptive information^49^ and nocebo responders showed increased activation in the thalamus compared to non-responders^50^. At first glance, our finding could also be interpreted as an artifact of the thalamus simply encoding higher differences in pain perception between the nocebo and control condition on day 1, leading to higher effects on day 8, however, it has to be kept in mind that we already accounted for the effects on day 1. Thus, a higher nocebo effect on day 1 cannot explain the observed relationship. A more promising explanation is based on the thalamus being linked to conditioning processes regarding pain^51^. Thus, stronger activity in the thalamus could be interpreted as an indicator of a stronger conditioning effect that exerts influence on stimulus processing during the test phase or, in line with the concept of self-reinforcing feedback loops, a stronger learning process from expectation-consistent information^8,9^. The thalamus therefore appears to play an additional role next to stimulus processing in the longer-term maintenance of nocebo effects.

Even though we found no significant behavioral differences between the persistence of nocebo and placebo effects, we see different neural patterns predicting the persistence of these effects. This fits the results of our previous study^28^ with differential neural patterns for positive and negative expectations during pain perception. Additionally, this might indicate that positive and negative valences of expectations rely on differential mechanisms that support their up-keeping. Interestingly, while several areas are connected to placebo and nocebo effects during anticipation of pain and during pain processing^28^, out of these only few regions seem to have predictive power for the long-term persistence of these effects.

On day 8, we observed an increased theta-to-alpha EEG power for negative compared to positive expectations during the anticipation of pain. This is in line with previous findings that indicated a high relevance of the pre-stimulus low frequency oscillatory activity that allow for the subsequent modulation of the pain perception^29–31,47,52^. Lower frequency activity, whether altered by spontaneous fluctuations or pain-related expectations, has been shown to affect the pain perception, although the direction of this modulation was inconsistent over studies^29–31,52^. Nevertheless, this points towards an important role of alpha activity for the top-down signaling of expectations that ultimately lead to the modulation of bottom-up sensory information as observed in placebo and nocebo effects^29,53^. As we observed these changes in neural activity one week after the original induction of expectations, this further supports the notion that expectations are not transient but instead result in true, lasting changes in perception. We did not detect any differences in neural processing during the pain phase, which is consistent with prior research and might be due to a transition from the expectation signaling predominant in the anticipation phase to other processes more focused on the signaling of sensory information and prediction errors during pain perception^29,31^. Moreover, there were no significant differences between oscillatory power for both placebo and nocebo compared to control. One possible explanation is that changes in EEG activity were more subtle, in line with the less pronounced behavioral differences between these conditions compared to the contrast between placebo and nocebo.

Taken together, our findings show that the initial experience with a treatment can have long-lasting effects in the same and other contexts. This implies the need to reduce or at least reframe negative experiences in order for them not to impede future treatments. Conversely, this also means that positive experiences can have long-term consequences and should be harnessed. This raises important clinical questions. Notably, the persistence of nocebo effects could serve as one crucial gateway for the development of chronic pain. The emergence of chronic pain is hypothesized to follow a complex interaction of factors, gathered in an integrative psychobiological pain model proposed by Büchel^54^. This model synthesizes the most common approaches for understanding the emergence of chronic pain and underscores the pivotal role of expectations. Following this model, persisting alterations of pain perception by negative expectations (which could also be termed as a stable nocebo effect) could act as a significant driving force, accelerating a vicious cycle leading to chronic pain. However, our findings regarding the persistence of placebo effects suggest that the opposite could also occur, manifesting as a virtuous cycle. This underscores the importance of carefully handling the expectations of patients. The question further arises if the persistence of expectation effects on perception could be an important factor in other disorders. Persistent expectations could e.g. play a role in psychosis, in which the influence of expectations on perception has already been discussed^55^.

This study is not without limitations. Our cover story which was based on a sham BCI allowed for a trial-by-trial manipulation of expectations within participants and was thereby a strength of our procedure, but might also be a limiting factor for the generalization of our results to other placebo/nocebo paradigms. Moreover, the stability of expectation effects was only demonstrated for a relatively short time period. Future research should investigate the persistence of placebo and nocebo effects over longer time courses and should ideally incorporate multiple measurements to dissect the temporal trajectory of these effects. Then, the change from the MRI lab on day 1 to the EEG lab on day 8 demonstrated that expectation effects persisted relatively independent of the context in which these effects were induced. However, a direct comparison of fMRI activity between both days was therefore not possible and might be a target for future studies. Finally, it is further important to note the correlative nature of our predictive fMRI analyses. To investigate the causal relationship between fMRI activity on day 1 and behavioral outcomes on day 8, a larger sample size would be necessary.

Overall, our results show that both placebo and nocebo effects can remain stable over one week despite a change in treatment context, and that the stability of the individual effects were determined by distinct neural correlates for positive and negative expectations. Especially for positive expectations areas related to learning, affective processing, and top-down control appear to predict the strength of responses one week after the initial induction of expectations.

## Methods

### Participants

The final sample of the present study consisted of 41 healthy participants. Initially, 50 participants took part in the first session of the measurement and were included in the previous study^28^. Out of these, 42 participated in a follow-up appointment one week later. As preregistered (German Clinical Trials Register; ID: DRKS00026174), one participant had to be excluded for the lack of expectation effects on day 1, indicated by higher ratings for the placebo compared to the nocebo condition averaged over the entire day, leading to a final sample size of *N* = 41 (26 female; age: *M* = 25.6, *SD* = 3.6 years, range: 18–34 years). Volunteers were recruited via an online job platform. All participants were right-handed, had normal or corrected-to-normal vision, reported no neurological or psychiatric diseases, pain conditions, current medication, substance abuse, or pregnancy, and were non-smokers. They gave written informed consent and were compensated with 15 Euros per hour of participation. Analyses for fMRI data were performed additionally excluding 2 participants due to technical issues leading to incomplete measurements, leading to a final sample size of *N* = 39 for fMRI analyses. The study was approved by the local ethics committee (PV7170). We confirm that all research was performed in accordance with relevant guidelines and regulations.

### Procedure

#### Day 1

The detailed procedure on day 1 has been described elsewhere^28^. Scripts that specify the instructions used can be found in the supplements (see Supplementary Methods S1). In brief, positive, negative, or neutral pain-related expectations were induced using verbal instructions and a conditioning procedure. Participants were informed that they would receive real-time feedback in regard to their current pain sensitivity by means of a BCI (brain-computer-interface). Three images of a brain with the right primary somatosensory cortex highlighted in one of three different colors served as visual cues: A green stimulus represented a state of low pain sensitivity (placebo condition), a red stimulus represented a state of high pain sensitivity (nocebo condition), and a yellow stimulus represented an intermediate state in which no prediction was made (control condition). After a pain calibration (for details see below), participants were conditioned using 10 trials of the placebo and 10 trials of the nocebo condition. This phase was disguised as a calibration of the BCI algorithm to the individual participants. Without the participants knowledge, green cues were always followed by a less painful stimuli (VAS30), red cues were always followed by more painful stimuli (VAS70). After these steps, the test phase was carried out (see below). The pain calibration, conditioning phase and test phase were conducted inside an MRI scanner. For both days, the experiment was programmed using the Psychtoolbox3 (http://psychtoolbox.org/) for Matlab (Version R2021b; The MathWorks).

#### Day 8

Day 8 took place approximately one week later (actual distance between the two sessions: *M* = 7.5 days, *SD* = 1.7, range: 5–13 days, with a small deviation of the intended span of 7 days due to logistical limitations like the availability of participants or EEG laboratory capacity) and was conducted in an EEG lab. There were only two phases: a pain calibration phase and a test phase. Expectations were not further reinforced on this day, i.e., there was no verbal instruction phase and no conditioning procedure. Participants were only informed that the same algorithm to predict their current state of pain sensitivity as one week prior would be used. Rating responses were given by the participants using a standard keyboard. Instructions and ratings were presented on a monitor with a resolution of 1920 × 1080 at a viewing distance of approximately 100 cm. After the test phase, participants were asked to fill out questionnaires, were debriefed and paid.

#### Experimental phases

##### Pain calibration phase

A PATHWAY CHEPS (Contact Heat-Evoked Potential Simulator) thermode (https://www.medoc-web.com/pathway-model-cheps), was utilized for pain stimulation. This device has a rapid heating rate of 70 °C/s and a cooling rate of 40 °C/s and is capable of delivering heat stimuli in the range of 30 °C to 55 °C in less than 300 ms. In all phases of the experiment, the baseline temperature was set to 32 °C, and the rise and fall rates were set to 70 °C/s. We used an altered version of the pain calibration by Horing et al.^56^. During the pain calibration, the thermode head was attached to a location directly proximal to the volar mid-forearm. To desensitize the skin, subjects were pre-exposed to 4 brief heat stimuli starting at 42 °C, with each consecutive stimulus increasing by 0.5 °C, up to 43.5 °C. Subsequently, we used a probabilistic tracking procedure for pain threshold determination^57^, consisting of eight stimuli of 4 s rated by binary decision (painful or not painful). The temperature of each stimulus was decided by the rating of the stimulus presented before, with a higher temperature chosen when the stimulus before was rated as not painful and a lower temperature when the stimulus before was rated as painful. The final temperature was chosen as the pain threshold. On day 1, a linear regression was employed to determine individual temperatures corresponding to values of VAS30, VAS60 and VAS70 on a visual analog scale (VAS) from 0 (“no pain”) to 100 (“unbearable pain”) for each participant. On day 8, the same procedure was used to determine the temperature corresponding to VAS60 only. The mean calibrated temperature corresponding to VAS60 on day 1 was 45.48 °C (*SD* = 1.28 °C, *Min* = 42.4 °C, *Max* = 48.8 °C) and 45.94 °C on day 8 (*SD* = 1.59 °C, *Min* = 43.2 °C, *Max* = 50.7 °C).

##### Test phase

On both day 1 and day 8, a test phase of identical procedure was carried out. Participants were informed that they would now receive feedback on their current pain sensitivity from the BCI system on each trial, which could be either highly pain-sensitive (red; nocebo condition), less pain-sensitive (green; placebo condition), or no prediction would be made as our algorithm could not detect a clear-cut state (yellow; control condition). Trials were structured as follows: After being presented with the cue (green, red, or yellow) for 2 s, participants were asked to rate how painful they expected the next stimulus to be on a VAS ranging from 0 to 100 (expectation rating) while the cue was still presented on screen (4 s). Then, there was a pre-stimulus phase of 3.3 s in which a fixation cross was presented (anticipation phase). Next, independently of the cue color, participants always received a painful stimulus of a temperature corresponding to VAS60 for 4 s (pain phase). There were 30 cues of each condition followed by pain stimulation divided into three blocks, summing up to a total of 90 stimuli. The ITI was fully randomized between 2 and 7 s. The order of cues was pseudo-randomized with no more than two direct repetitions of the same condition, and there was a different trial order for day 1 and day 8. During the first block of the test phase, the thermode head was attached to a location directly proximal to the volar mid-forearm. The thermode position was changed to a position directly distal to the volar mid-forearm for the second block and then back to the original position in the third block. Before each block, we applied one pain stimulus of VAS60 without a cue to desensitize the new skin area.

### Data acquisition

#### EEG data

On day 8, continuous EEG data was recorded inside an electrically shielded room using a 64-channel actiCAP and the BrainVision Recorder (BrainProducts, Gilching, Germany). The cap contained 64 active Ag/AgCl electrodes with 62 electrodes arranged according to the extended 10/20 System and the two remaining electrodes used to record a bipolar horizontal electrooculogram (HEOG). FCz served as reference and Pz served as ground electrode. The cap was connected to two BrainAmp amplifiers with 32 channels each (BrainProducts, Gilching, Germany), powered by rechargeable battery units. Electrode skin impedance was kept below 20 kΩ. EEG data was recorded with a sampling rate of 500 Hz and an amplitude resolution of 0.1 µV. Data was filtered online with a low cut-off filter with a time constant of 10 s and a high cut-off at 1,000 Hz.

#### fMRI data

For MRI measurements on day 1, a 3T Siemens PRISMA Scanner equipped with a 64-channel head coil was utilized. For the experiment, two sequences were acquired: an EPI BOLD sequence and a field map sequence. Participants were equipped with a 64-channel standard BrainCap MR for 3 Tesla (2020 Version). The EPI BOLD sequence parameters included: TE: 29.0 ms, TR: 1679.00 ms, FOV: 22.4 * 22.4 cm, flip angle: 70°, slice thickness: 2 mm, scan time: 20 min and 17 s, and a total of 715 volumes acquired. One day prior, a T1 image with the following parameters was acquired: T1 FLASH 3D: TE 2.98 ms, TR: 2.3 s, matrix flip angle: 9°, FOV 25.6 * 25.6 cm, TA: 7:22 min.

### Preprocessing

#### EEG data

The Fieldtrip toolbox for Matlab^58^ was used for the preprocessing of EEG data. The data were segmented into trials from 1,000 ms prior to cue onset to the end of pain stimulation 15,800 ms following cue onset. The resulting segmented data were filtered (low-pass filter at 150 Hz, high-pass filter at 0.5 Hz) We adapted a recently introduced preprocessing approach (see Hipp et al.^59^). To obtain maximal sensitivity in detecting and removing artifacts, the data was split into low- and high-frequency data (34 Hz low-pass filter and 16 Hz high-pass filter, respectively) and processed in parallel. All single trials were visually inspected and removed for both high- and low-frequency data when containing excessive artifacts. Subsequently, both subsets underwent independent component analysis (ICA) using a logistic infomax algorithm. Components reflecting e.g., blinks, eye- and head movement, or muscle activity were visually identified based on the time course, spectrum, and topography and discarded. Both subsets were re-referenced to the average of all EEG channels and the original reference electrode (FCz) was regained. Finally, all data were subjected to another comprehensive visual scan, and the time axis was adjusted to align with the onset of pain stimulation at t = 0 s. The visual artifact screening process led to the exclusion of 97 of the 3690 recorded trials (2.63%) in total.

##### Time-frequency decomposition

Time-frequency decomposition was conducted for 21 logarithmically spaced frequencies ranging from 4 to 128 Hz (0.25-octave increments) in 0.1 s steps using the multi-taper method based on discrete prolate spheroid sequences (DPSS), adapted from Hipp et al.^59^. For the frequency transformation of frequencies above 25 Hz, high frequency data were used, while for frequencies below 25 Hz, low frequency data were used. Temporal and spectral smoothing were adjusted to match 250 ms and 3/4 octave, respectively, by fixing the time window to 250 ms and adjusting the number of Slepian tapers for frequencies above 16 Hz and using a single taper but adjusting the time window for frequencies up to 16 Hz. The single-trial time-frequency resolved data were averaged per condition for each participant.

#### fMRI data

Preprocessing of fMRI data was done using the Statistical Parameter Mapping software (SPM 12, Wellcome Department of Imaging Neuroscience, London, UK, https://www.fil.ion.ucl.ac.uk/spm/software/spm12/). The first two volumes of each block were dropped to get full MRI saturation effects and data then underwent realignment and unwarping, registration to standard space (Montreal Neurological Institute), and spatial smoothing with a 6 mm Gaussian kernel.

### Data analysis

#### Behavioral data

We compared differences in pain and expectation ratings for the different cue conditions at two different time points by computing two-way repeated measures ANOVAs with pain and expectation ratings as outcomes, respectively, and cue type (placebo vs. nocebo vs. control) and session (day 1 vs. day 8) as predictors. Moreover, we compared the relative variability index^60^ between conditions using two rmANOVAs with cue type (placebo vs. nocebo vs. control) and session (day 1 vs. day 8) as predictors and the relative variability index of expectation and pain ratings as outcomes, respectively, to determine if the variability in individual ratings differed between conditions and days. For the remaining analyses, we computed the placebo effect (control - placebo) and nocebo effect (nocebo - control) in a way to ensure that positive values always mean higher modulation of ratings in the intended direction. Rating modulations induced by placebo and nocebo effects at the two different time points were examined by computing rmANOVAs for expectation and pain ratings separately, using (1) modulation type (placebo effect vs. nocebo effect) and time point (day 1 vs. day 8), and (2) modulation type (placebo effect vs. nocebo effect) and time point (last 5 trials of each condition on day 1 vs. first 5 trials of each condition on day 8) as predictors. Significant effects in all ANOVAs were followed up by conducting Bonferroni-Holm corrected post-hoc *t*-tests. To assess predictors of the rating modulation on day 8, we correlated the rating modulation during day 1 with the modulation during day 8 (placebo effect with placebo effect, nocebo effect with nocebo effect), separately for expectation and pain ratings. To explore whether the duration of the inter-test period affected the stability of placebo and nocebo effects, we correlated the individual inter-test period with placebo and nocebo effects on day 8 and with the absolute change in placebo and nocebo effects from day 1 to day 8 (e.g. placebo effect on day 8 minus placebo effect on day 1), for both pain and expectation ratings.

#### EEG data

We compared differences in power between (1) the placebo and nocebo condition, (2) the placebo and control condition, and (3) the nocebo and control condition in the anticipation phase and pain phase separately at each time-frequency-electrode point for high- and low-frequency data. We statistically tested power differences using nonparametric cluster-based permutation tests as implemented in the Fieldtrip toolbox (cluster threshold: *p* = .05, minimum neighbors: 2, number of randomizations: 2000). Statistics were calculated in the time range of -3.3 until − 0.1 s for the anticipation phase and 0 until 3.9 s for the pain phase.

#### fMRI data

##### Statistical inference

Identical to our previous publication^28^, for each subject, a Finite Impulse Response model (FIR model) was set up on a time course of 18.4 s starting at the onset of the cue, divided into 11 bins, with a bin roughly covering the duration of one TR (1.679 s compared to 1.675 s). The FIR model was implemented separately for each condition. Data was also corrected for cardioballistic and respiratory artifacts by including them as regressors built with the RETROICOR algorithm of the PhysIO toolbox^61,62^.

Contrasts were formed on the first level comparing placebo to control and nocebo to control separately in the anticipation and pain phase. Analyses during the anticipation phase were conducted by using FIR regressors that spanned the period from − 4.275 s to − 0.925 s before the onset of pain (corresponding to bins 4 and 5). For the pain phase, the analyses utilized FIR regressors covering the interval from 0.75 s to 5.8 s after pain onset (corresponding to bins 7, 8, and 9). The contrasts were then used in one-sample *t*-tests on the second level including two covariates. For placebo-related fMRI activity (placebo vs. control), one covariate was formed from the behavioral placebo effects from day 8 to capture the stability of the effects between the two measurements and added on the second position of the design matrix. To assure that only the effects of day 8 had an influence on the brain activity, the placebo effect of day 1 was inserted as a covariate on the first position of the design matrix, assuring an orthogonalization of the vectors and thus only the variance that should go beyond the effect of day 1 remains. Conversely, for nocebo-related fMRI activity (nocebo vs. control), the behavioral nocebo effect on day 1 was included in the first position of the design matrix as a covariate, while the nocebo effect on day 8 was included in the second position. This was done separately for the anticipation and the pain phase. Analyses were corrected for multiple comparisons using FWE correction (*p* < .05).

#### ROI analyses

Adapted from Wittkamp & Wolf et al.^28^, ROI analyses were conducted in the following areas defined by the anatomy based on the Harvard-Oxford atlas: insular cortex, thalamus, ACC, hippocampus and amygdala. Additionally, a ROI analysis was conducted on the DLPFC based on the clusters identified in the meta-analysis conducted by Zunhammer et al.^27^ by applying a 15 mm-radius sphere around the two reported peak coordinates (xyz_MNI_: 42, 11, 33 and xyz_MNI_: -30, 13, 54) bilaterally. Further, a ROI analysis on the angular gyrus was conducted based on the results of Wittkamp & Wolf et al.^28^ with a 15 mm-radius sphere around the peak coordinate during anticipation of pain (xyz_MNI_: 56, -54, 40) and during pain perception (xyz_MNI_: 44, -50, 38).

## Electronic Supplementary Material

Below is the link to the electronic supplementary material.

Supplementary Material 1

## Data Availability

Derived data that support the findings of this study are available at https://osf.io/r5ejs/. For additional information and requests, please contact Michael Rose directly.
