# Supplementary material for: Differential neural activity predicts the long-term stability of the effects of positive and negative expectations on pain
Source: Sci Rep. 2024 Nov 13;14:27874. doi: 10.1038/s41598-024-77693-z (PMC11561249; doi:10.1038/s41598-024-77693-z)
Supplement: Supplementary file 1 — Supplementary Material 1 [file 41598_2024_77693_MOESM1_ESM.pdf]

SUPPLEMENTARY MATERIAL

Differential neural activity predicts the long-term stability of the effects of positive and negative expectations on pain

Maren-Isabel Wolf[1], Christoph Arne Wittkamp[1], Michael Rose*

Department of Systems Neuroscience, University Medical Center Hamburg-Eppendorf, Germany

[1] These authors contributed equally to this work.

* Corresponding author

Cover story – originally given in German

1. **Verbal Instructions**

These instructions were given in a participant preparation room before the scan. The images shown were presented on a screen.

Slide 1

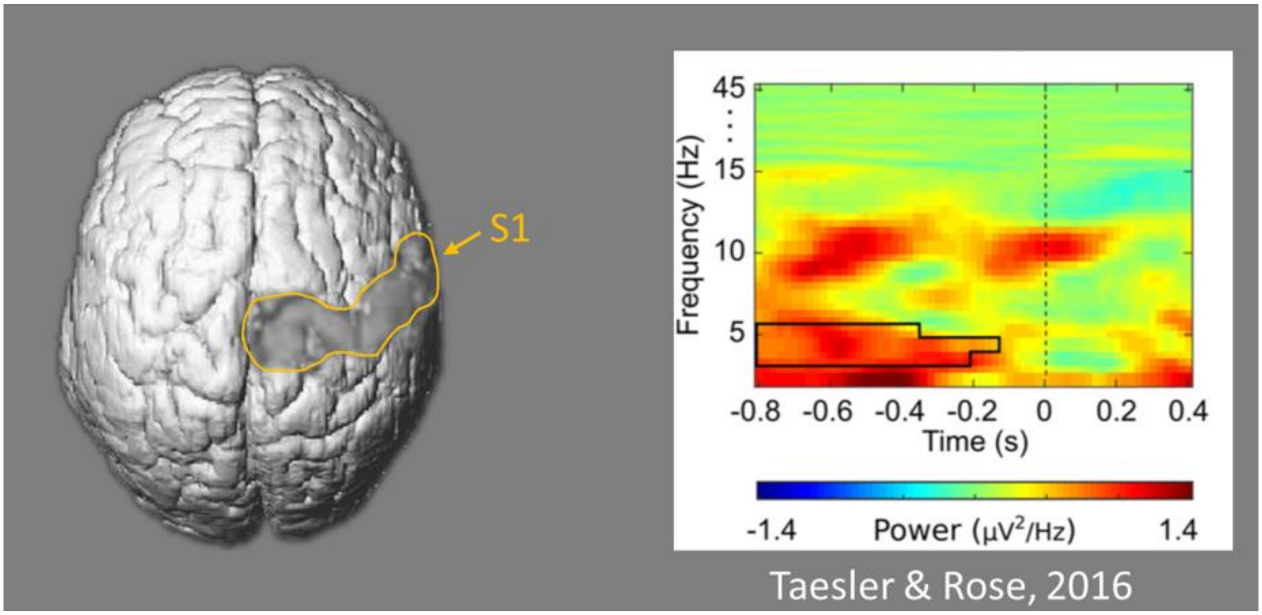

- In this experiment, we will administer pain stimuli to the arm and measure brain states using electroencephalogram (EEG).
- Now, we will briefly explain the basics of the experiment. These do not need to be understood in detail, we only want to establish a fundamental understanding.
- Previous research, also from our institute, shows interesting findings: equally strong pain stimuli were perceived differently in studies, which seems to depend on the brain state prior to the pain

stimulus. If a brain area responsible for sensory processing fires in a different pattern, participants experienced more intense pain. We want to investigate this again today.

- *Left Image*
    - Particularly important: somatosensory cortex → *Show position of S1*.
- *Right Image*
    - Brain state = activity in certain frequency ranges, faster or slower "firing" of neurons.
    - Can be indicated in frequency range (*show scale*).
    - Taesler & Rose (2016): a study from our institute, equally strong pain stimuli were either perceived as painful or not; characteristic activity before pain stimulus when perceived as painful.
    - Theta waves marked = 4-8 Hz, there was strong theta activity in the somatosensory cortex when the perception was painful.
    - In this study, we will attempt to predict in real-time how much pain the following stimulus will cause you based on the currently measured brain waves.

Slide 2

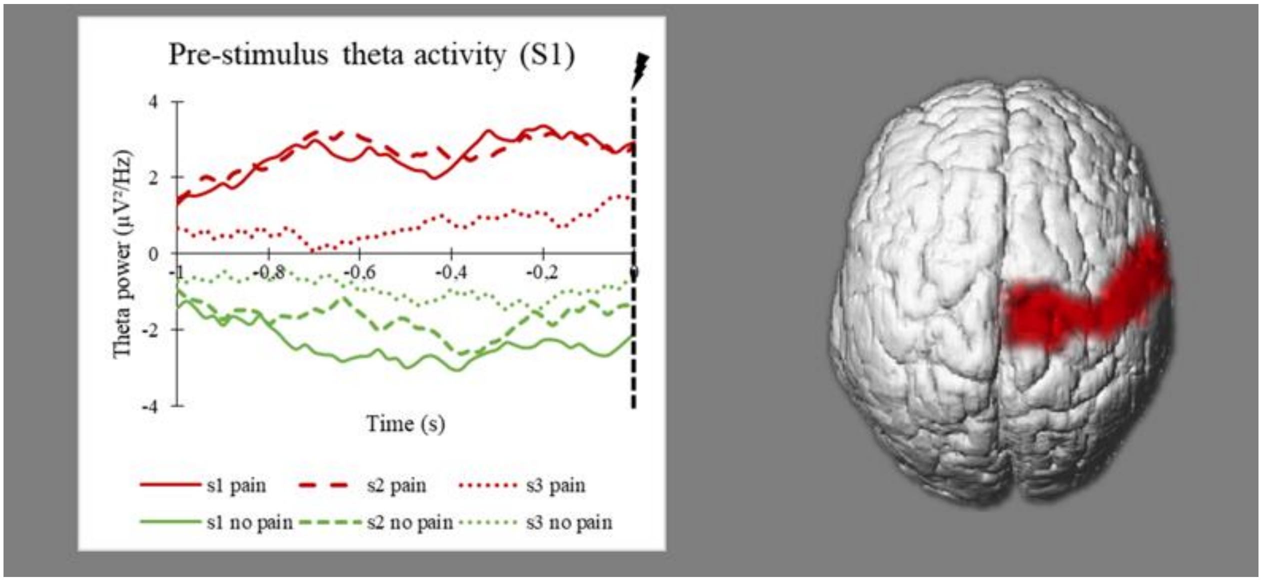

- *Left Image*
    - Here, you can see data from three participants from the last study.
    - The plot shows the time course of theta activity in the somatosensory cortex before equally strong pain stimuli, which were either perceived as painful (red curves) or not painful (green curves).
    - Red line = strong theta activity when the stimulus was later perceived as painful.
- *Right Image*
    - If we measure such a state of high pain sensitivity, as can be seen with the red curves, in you, you will be shown this brain image with the red coloring of the corresponding area.

Slide 3

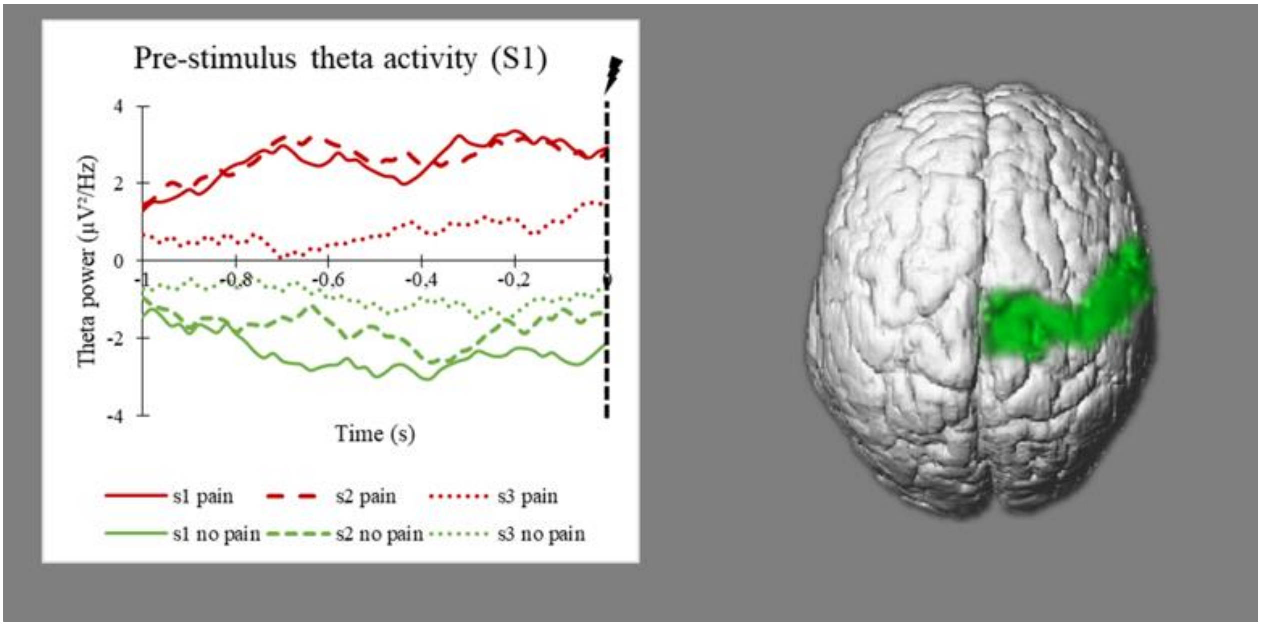

- *Left Image*
  – Green line = weak theta activity when the stimulus was later perceived as not painful.
- *Right Image*
  – If we measure such a state of low pain sensitivity in you, you will be shown this brain image with the green coloring of the corresponding area

Slide 4

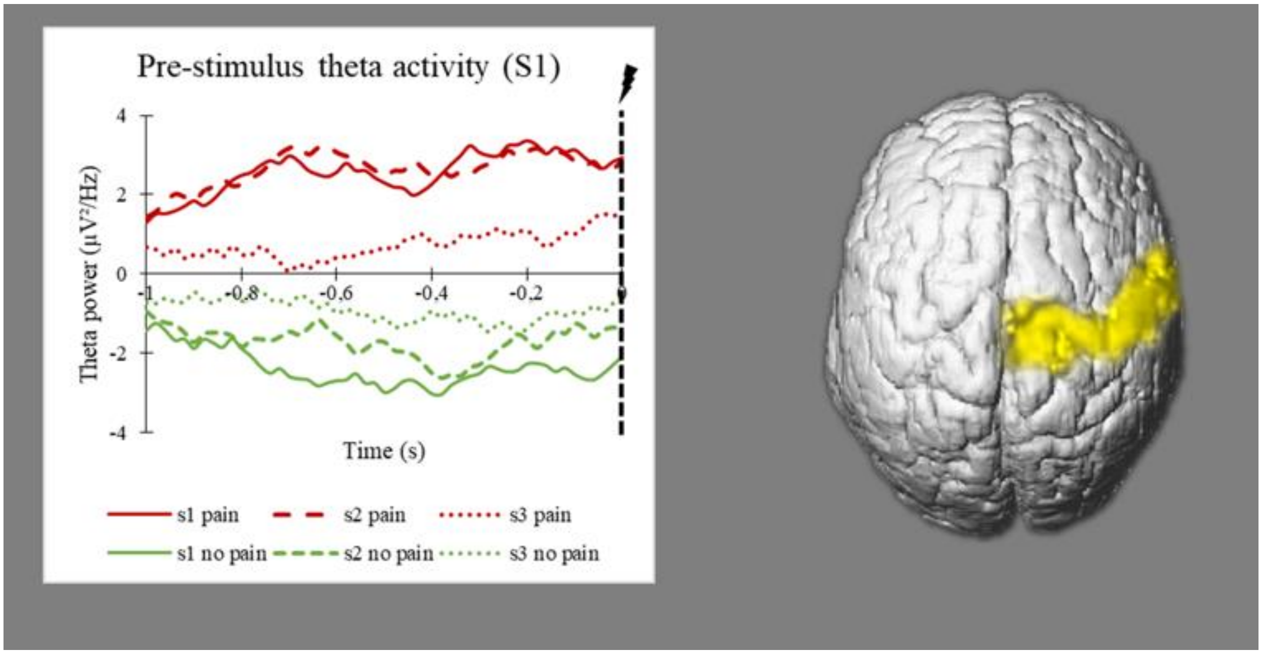

- If our algorithm can't accurately detect a clear brain state before the pain stimulus (e.g. because the activation is in the middle, fluctuates too much, or our measurement is incorrect), the area is colored yellow.

Slide 5

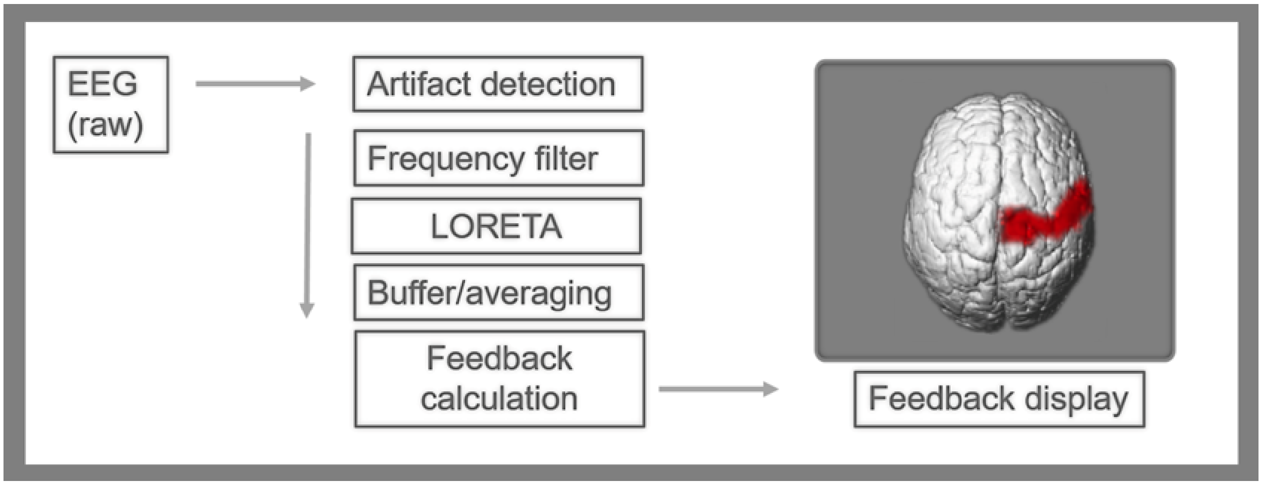

- We will do a live measurement. To calculate the current state, we use a Brain-Computer Interface (BCI) algorithm, which we use to determine the current theta activity.
- *Image*
    - The brain signal is processed in several stages:
      1. EEG signal is captured.
      2. Correction of disturbing artifacts, as shown earlier.
      3. Filtering out frequencies that are not of interest to us.
      4. Source localization/position of the signal.
      5. Averaging.
      6. Visual feedback.
- The objective of the study is to evaluate the algorithm and replicate previous findings.

2. Conditioning

These instructions were given directly before the conditioning phase, with the participant already positioned in the scanner.

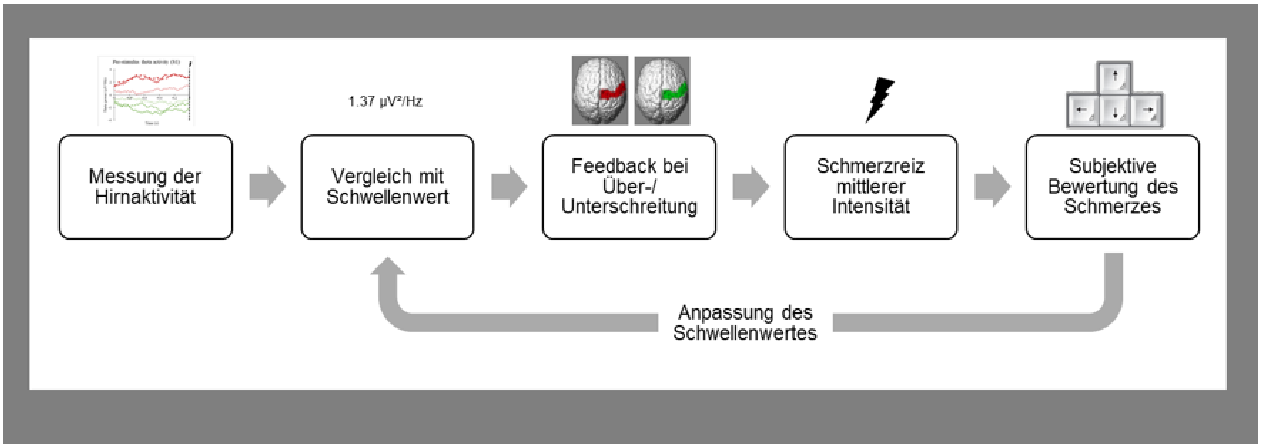

- A calibration of the system is necessary as the threshold between brain states varies between persons.
- *Image*
    - Process:
      1. Start with a typical population threshold.

2. Exceeding/Undershooting of the threshold → A pain stimulus of ~ medium intensity will be sent.
3. Pain assessment on a scale (arrow keys left/right).
4. With this feedback, we will adjust the threshold → gradual adjustment of the algorithm, not of the pain stimuli; you will hardly notice this.
5. There will be no yellow feedback; the stimulus is only sent if the threshold is clearly exceeded or undershot.

- At the end, we will check whether the threshold was determined sufficiently accurately.

3. Test phase

These instructions were given directly before the test phase on day 1 and there was a short repetition of these instructions on day 8.

- Pain stimuli will be presented at specific time points.
- Pain stimuli will be different stimuli of medium intensity.
- You will receive feedback on whether the brain state is clearly above or below the threshold or if the algorithm does not make a prediction (yellow).
- The yellow marked state is now also possible because we do not wait for over/under-threshold activity.
